# 新诊断多发性骨髓瘤自体造血干细胞移植后达雷妥尤单抗维持治疗的临床观察

**DOI:** 10.3760/cma.j.issn.0253-2727.2023.12.008

**Published:** 2023-12

**Authors:** 艺 马, 秀斌 肖, 喜林 陈, 顺宗 袁, 云 鲁, 世华 赵, 俊丽 陈, 广宁 石, 玥琦 王, 娜娜 程, 盼 封, 明爽 丁, 文荣 黄

**Affiliations:** 解放军总医院第五医学中心血液病医学部淋巴瘤-浆细胞疾病专科，北京 100071 Department of Lymphoma & Plasma Cell Disease, Senior Department of Hematology, the Fifth Medical Center of PLA General Hospital, Beijing 100071, China

**Keywords:** 多发性骨髓瘤, 达雷妥尤单抗, 造血干细胞移植, 维持治疗, Multiple myeloma, Daratumumab, Autologous stem cell transplantation, Maintenance therapy

## Abstract

**目的:**

探讨新诊断多发性骨髓瘤（NDMM）患者自体造血干细胞移植（auto-HSCT）后达雷妥尤单抗维持治疗的疗效及安全性。

**方法:**

回顾性分析2022年5月1日至2023年6月30日期间解放军总医院第五医学中心血液病医学部淋巴瘤-浆细胞疾病专科的15例NDMM患者auto-HSCT后接受达雷妥尤单抗维持治疗的临床资料、血液学及肾脏疗效、安全性。

**结果:**

15例患者中男11例，女4例，中位年龄58（41～72）岁；其中13例患者于诱导治疗及auto-HSCT阶段均未使用达雷妥尤单抗，6例伴有肾损害，9例遗传学高危。达雷妥尤单抗维持治疗的中位时间为6（1.5～12）个月，中位输注次数12（6～17）次。15例患者均可评估疗效，达雷妥尤单抗维持治疗使缓解深度增加，血液学严格意义的完全缓解率（sCR）由40％增加至60％；6例伴肾损害患者的肾脏缓解率由16.67％ 提升至33.33％。血液学3级以上不良事件（AE）主要为淋巴细胞减少（26.67％，4/15），非血液学AE主要为输注相关反应（46.67％，7/15）及3级肺炎（33.33％，5/15）。5例（38.46％，5/13）发生肺炎的患者均为达雷妥尤单抗初治者，发生时间在中位输注8（6～10）次，3例肺炎为肺间质改变，甲泼尼龙治疗有效。中位随访时间为12（7～13）个月，1例死亡（与达雷妥尤单抗治疗无关），1年总生存率为93.33％。

**结论:**

达雷妥尤单抗用于NDMM患者auto-HSCT后维持治疗可提高血液学及肾脏缓解率，且不良反应可控。主要的AE是3级肺炎，减少维持治疗期间前8周每周一次给药频次可能使肺炎发生率下降。

## Abstract (en)

**Objective:**

This study aimed to evaluate the efficacy and safety of daratumumab as a maintenance treatment after autologous hematopoietic stem cell transplantation（auto-HSCT）in patients with newly diagnosed multiple myeloma（NDMM）.

**Methods:**

The clinical data, hematological and renal response, and safety of 15 post-transplant patients with NDMM who had received daratumumab maintenance between May 1, 2022 and June 30, 2023 were retrospectively analyzed.

**Results:**

Fifteen patients（11 males and 4 females）with a median age of 58（41−72）years were included. Thirteen patients did not receive daratumumab during induction therapy and auto-HSCT, 6 patients had renal impairment, and nine patients had high-risk cytogenetics. The median infusion of daratumumab was 12（6−17）times, and the median duration of maintenance was 6（1.5−12）months. The treatment efficacy was evaluated in all 15 patients, and daratumumab maintenance therapy increased the rate of stringent complete response from 40％ to 60％. The renal response rate and median estimated glomerular filtration rate of six patients with RI-NDMM were also improved. During daratumumab maintenance therapy, the most common hematological grade 3 adverse event（AE）was lymphopenia［4 of 15 patients（26.67％）］, whereas the most common nonhematologic AEs were infusion-related reactions［7 of 15 patients（46.67％）］and grade 3 pneumonia［5 of 15 patients（33.33％）］. The five patients with pneumonia were daratumumab naive［5 of 13 patients（38.46％）］, with a median of 8（6−10）infusions. Among them, the chest computed tomography of three patients showed interstitial infiltrates, and treatment with methylprednisolone was effective. With a median follow-up of 12 months, the 1-year overall survival rate was 93.33％, and only one patient died（which was not related to daratumumab treatment）.

**Conclusion:**

Daratumumab was safe and effective as a maintenance agent for post-auto-HSCT patients with NDMM, and AEs were controllable. The most common nonhematologic AE was grade 3 pneumonia, and a less dose-intense maintenance regimen for the first 8 weeks could reduce the incidence of pneumonia.

多发性骨髓瘤（MM）是以浆细胞克隆性增殖为特点的血液系统恶性肿瘤，随着自体造血干细胞移植（auto-HSCT）成为适合移植患者的一线推荐治疗，以及蛋白酶体抑制剂、免疫调节剂的引入，10年生存率可达60％[1]，但是MM具有不可治愈性，终将面临复发风险。新诊断多发性骨髓瘤（NDMM）在auto-HSCT后维持治疗可进一步提高缓解率，改善生存。来那度胺是auto-HSCT后维持治疗的最常用药物，但耐药性不可避免，且部分患者因不良反应而较难长期服用，因此仍不能满足维持治疗需求。达雷妥尤单抗是靶向CD38的IgG1-κ单克隆抗体，已有前瞻性研究显示其维持治疗的优势[2]，而我国关于达雷妥尤单抗维持治疗的数据有限。本研究回顾性分析了我中心15例NDMM患者auto-HSCT后达雷妥尤单抗维持治疗的有效性及安全性，现报告如下。

## 病例与方法

一、病例

回顾性分析2022年5月1日至2023年6月30日期间auto-HSCT后接受达雷妥尤单抗维持治疗的解放军总医院第五医学中心血液病医学部淋巴瘤-浆细胞疾病专科的15例NDMM患者临床资料。骨髓检查包括骨髓涂片、多参数流式细胞术（MFC）检测微小残留病（MRD）、骨髓活检病理及经CD138分选FISH技术检测细胞遗传学［del（13q）、del（17p）、t（4；14）、t（11；14）、t（14；16）、t（14；20）、1q21扩增］，MFC检测MRD的敏感度为10^−4^，MM的诊断依据IMWG标准[3]。梅奥诊所定义[4]高危细胞遗传学异常指FISH技术检测出del（17p）、t（4；14）、t（14；16）、t（14；20）、1q21扩增或p53突变，双打击MM：存在任意2个高危细胞遗传学异常。

二、治疗

1. 维持治疗方案：NDMM患者于auto-HSCT后2～3个月且血小板计数恢复至80×10^9^/L、白细胞计数恢复至3.0×10^9^/L以上者接受维持治疗。未曾应用达雷妥尤单抗者维持治疗：达雷妥尤单抗16 mg/kg，第1～8周，每周一次；第9～12周，每2周一次，此后每4周一次；有达雷妥尤单抗治疗史者维持治疗：达雷妥尤单抗16 mg/kg，每2周一次，连续8次，此后每4周一次。疗效评价每3个月一次，停药指征：病情进展或不可耐受不良反应、严重并发症，或患者不愿继续用药。

2. 输注相关反应（IRRs）预防：用药前给予地塞米松20 mg，非甾体类及抗组胺药物。

3. 病毒再激活预防：自维持治疗开始至治疗结束6个月，口服阿昔洛韦预防带状疱疹病毒激活；口服复方新诺明（合并肾功能不全者除外）预防卡氏肺囊虫肺炎；监测HBV-DNA及肝功能，乙肝表面抗原阳性者口服恩替卡韦抗病毒治疗。

三、疗效标准及安全性评价

血液学疗效评价采用IMWG标准[3]，分为严格意义的完全缓解（sCR）、完全缓解（CR）、非常好的部分缓解（VGPR）、部分缓解（PR）、微小缓解（MR）、疾病稳定（SD）、疾病进展（PD）。肾脏疗效评价亦采用IMWG标准[5]，分为肾脏CR：估算肾小球滤过率（eGFR）持续改善（至少2个月以上），从<50 ml·min^−1^（1.73m^2^）^−1^改善至≥60 ml·min^−1^（1.73m^2^）^−1^；肾脏PR：eGFR持续改善，从<15 ml·min^−1^·（1.73m^2^）^−1^上升至30～59 ml·min^−1^（1.73m^2^）^−1^；肾脏MR：eGFR持续改善，从<15 ml·min^−1^（1.73m^2^）^−1^提高至15～29 ml·min^−1^（1.73m^2^）^−1^，或从15～29 ml·min^−1^（1.73m^2^）^−1^提高至30～59 ml·min^−1^（1.73m^2^）^−1^。安全性评价：依照美国常见不良反应术语评定标准5.0版分级标准评定。

四、随访

通过查阅住院病历、门诊病历及电话随访，随访截止日期为2023年6月30日，中位随访时间 12（7～13）个月。无进展生存（PFS）期定义为自达雷妥尤单抗开始至任何原因导致疾病进展或死亡的时间，总生存（OS）期定义为自达雷妥尤单抗开始至任何原因导致死亡的时间。

五、统计学处理

采用Graphpad Pism 9.5统计学软件进行统计分析，使用*M*（范围）进行数据统计，组间比较采用Wlicoxon符号秩和检验进行差异分析，*P*<0.05为差异具有统计学意义。

## 结果

1. 一般资料：入组15例患者，男女比例11: 4，中位年龄58（41～72）岁；其中6例（40.0％）伴有肾损害（renal impairment，RI），该6例患者移植后eGFR见表1；9例（60.0％）为遗传学高危，包括：t（4；14）1例，1q21扩增8例（53.33％），8例1q21扩增者中包含1例双打击［del（17p）并1q21扩增］。14例患者移植后血液学疗效≥PR，1例SD；6例RI-NDMM移植后肾脏疗效均≥MR（表1）。15例患者中13例（86.67％）为达雷妥尤单抗初治，即诱导治疗及auto-HSCT阶段均未使用达雷妥尤单抗。该15例患者诱导治疗方案包括：Vd（硼替佐米/地塞米松）、VCd（硼替佐米/环磷酰胺/地塞米松）、VRd（硼替佐米/来那度胺/地塞米松）、VPd（硼替佐米/泊马度胺/地塞米松）、KPd（卡非佐米/泊马度胺/地塞米松）、Vd-PACE（硼替佐米/地塞米松/卡铂/表柔比星/环磷酰胺/依托泊苷）、Dara-Vd（达雷妥尤单抗/硼替佐米/地塞米松）、Dara-Vd-PACE，诱导治疗中位疗程数为6个。该15例患者auto-HSCT后接受达雷妥尤单抗维持治疗，达雷妥尤单抗中位输注次数12（6～17）次，中位维持治疗时间6（1.5～12）个月。

**表1:** 15例新诊断多发性骨髓瘤患者基本特征及诱导治疗后与移植后、维持治疗后疗效

例号	年龄（岁）	性别	分型	细胞遗传学	DS分期	ISS分期	eGFR[ml·min^−1^·（1.73m^2^）^−1^]	血液疗效			肾脏疗效		
								诱导后	移植后	维持后	诱导后	移植后	维持后
1	58	男	IgG-λ	标危	ⅢB	Ⅲ	22.94	SD	SD	CR	MR	MR	MR
2	68	男	κ	高危	ⅢB	Ⅲ	30.00	PR	sCR	sCR	MR	PR	PR
3	66	男	λ	标危	ⅡB	Ⅲ	15.94	CR	CR	sCR	MR	MR	PR
4	57	女	IgA-κ	高危	ⅢB	Ⅲ	27.20	CR	CR	CR	MR	MR	MR
5	60	男	λ	高危	ⅡB	Ⅲ	54.21	sCR	sCR	sCR	MR	MR	MR
6	49	女	IgA-κ	标危	ⅡB	Ⅲ	36.47	PD	PR	VGPR	MR	MR	MR
7	59	男	λ	高危	ⅡA	Ⅰ	108.00	PD	VGPR	sCR	−	−	−
8	54	女	IgG-κ	高危	ⅢA	Ⅲ	162.00	VGPR	VGPR	sCR	−	−	−
9	46	男	IgG-λ	标危	ⅢA	Ⅱ	149.00	CR	sCR	sCR	−	−	−
10	53	男	IgG-κ	高危	ⅡA	Ⅱ	133.00	CR	CR	CR	−	−	−
11	56	女	IgA-κ	高危	ⅢA	Ⅲ	157.00	sCR	sCR	sCR	−	−	−
12	62	男	κ	标危	ⅢA	Ⅰ	175.00	CR	sCR	sCR	−	−	−
13	41	男	IgA-κ	高危	ⅢA	Ⅱ	164.00	CR	CR	CR	−	−	−
14	62	男	IgA-κ	高危	ⅢA	Ⅲ	137.00	CR	CR	CR	−	−	−
15	72	男	κ	标危	ⅢA	Ⅰ	130.00	CR	sCR	sCR	−	−	−

注 细胞遗传学高危：FISH检测出：t（4;14）、t（14;16）、t（14;20）、del（17p）、1q21扩增或p53突变；细胞遗传学标危：细胞遗传学高危之外的所有其他类型，包括：三倍体、t（11;14）、t（6;14）；DS: Durie-Salmon分期体系；ISS：国际分期体系；eGFR：估算肾小球滤过率；SD：疾病稳定；CR：完全缓解；sCR：严格意义的完全缓解；PR：部分缓解；VGPD：非常好的部分缓解；PD：疾病进展；MR：微小缓解；−：不适用

2. 不良事件（AE）：血液学AE：3级血红蛋白减少、血小板减少及白细胞减少各1例，3级淋巴细胞减少4例（26.67％，4/15），未发生4级血液学AE。非血液学AE：主要为IRRs、低免疫球蛋白血症、肺炎。7例（46.67％，7/15）发生IRRs，均为1～2级，其中6例发生于首次输注，1例发生于第2次输注。15例患者IgG、IgA及IgM水平均有不同程度下降，排除未达CR患者血液中M蛋白干扰，观察诱导治疗后血液学疗效CR的10例患者IgG、IgA及IgM水平，可见三者的中位值于维持治疗前即低于正常，维持治疗使其进一步降低，IgG及IgA中位值较维持治疗前差异具有统计学意义（*P*＝0.004，*P*＝0.002）（表2）。3级肺炎5例（33.33％，5/15），均为达雷妥尤单抗初治者，初治者肺炎比例为38.46％（5/13），肺炎发生于达雷妥尤单抗中位输注8（6～10）次时。痰标本及肺泡灌洗液标本经微生物培养及二代测序检测，检出致病微生物：肺炎克雷伯菌、嗜麦芽窄食单胞菌、近平滑念珠菌、EB病毒、冠状病毒OC43。3例肺炎患者肺部CT提示肺间质改变，临床表现为发热、咳嗽、胸闷气喘等症状，抗细菌、真菌或病毒等治疗使呼吸道症状部分缓解或无明显缓解，联合甲泼尼龙治疗后肺炎吸收。1例肺炎因并发心功能不全而终止达雷妥尤单抗治疗；其余4例肺炎痊愈后继续每4周一次输注，未再发生肺炎。

**表2:** 10例诱导治疗后完全缓解患者不同治疗阶段免疫球蛋白水平（中位值，g/L）

时间	IgG	IgA	IgM
诱导治疗后	5.480	0.480^a^	0.360
自体造血干细胞移植后	6.200	0.190	0.220
达雷妥尤单抗维持治疗后	5.120^a^	0.050^a^	0.190

注 免疫球蛋白正常参考值范围：IgG 7.00～16.00 g/L, IgA 0.70～4.00 g/L，IgM 0.40～2.30 g/L；^a^ 与达雷妥尤单抗维持治疗前相比，*P*<0.05

2例患者于达雷妥尤单抗治疗后2个月感染新型冠状病毒，1例并发多脏器功能衰竭死亡。4例HBcAb 阳性，随访期间监测HBV-DNA均为阴性，且未发生肝功能异常；15例患者均未发生带状疱疹；6例RI-NDMM均未发生肾损害加重。

3. 疗效评价：①血液学疗效：1例SD转为CR，1例PR转为VGPR，2例VGPR（均为1q21扩增）转为sCR，总体sCR率由40.0％（6/15）提升至 60.0％（9/15），MRD阴性率由40.0％（6/15）提升至53.33％（8/15）。遗传学高危者疗效：8例1q21扩增者sCR率由25.0％（2/8）提升至50.0％（4/8），1例双打击及1例t（4；14）患者均保持维持治疗前CR及sCR疗效，遗传学高危者总体sCR率由33.33％（3/9）提升至55.56％（5/9）（表1）。②肾脏疗效：6例RI-NDMM患者，4例为重度RI［eGFR<30 ml·min^−1^·（1.73m^2^）^−1^］，达雷妥尤单抗中位输注12（10～17）次。1例MR转为PR，中位eGFR提升了24.37％［28.60 ml·min^−1^·（1.73m^2^）^−1^对35.57 ml·min^−1^·（1.73m^2^）^−1^］。例3为auto-HSCT后脱离透析者，输注达雷妥尤单抗10次，eGFR提升了91.03％［15.94 ml·min^−1^·（1.73m^2^）^−1^对30.45 ml·min^−1^·（1.73m^2^）^−1^］。6例RI-NDMM患者auto-HSCT前及达雷妥尤单抗输注10次前后eGFR对比见图1。

**图1:**
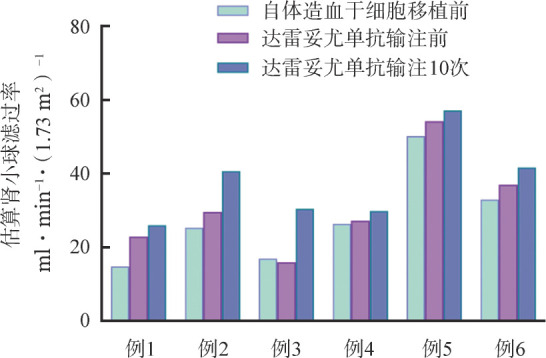
6例伴肾损害患者移植前及达雷妥尤单抗输注10次前后估算肾小球滤过率对比图

4. 生存分析：中位随访12（7～13）个月，中位PFS及OS期未达到，1例死亡（感染新型冠状病毒并发多脏器功能衰竭），1年OS率为93.33％。至随访期结束，5例患者继续达雷妥尤单抗维持治疗。

## 讨论

MM是不可治愈的恶性单克隆浆细胞病，即使处于新药时代，复发仍不可避免，而维持治疗是MM全程治疗中延缓复发的重要环节。CASSIOPEIA[2]及GRIFFIN[6]两项前瞻性研究表明达雷妥尤单抗可以作为适合移植的MM患者的有效维持治疗手段，但需要更多的研究来验证其在真实世界中的疗效及安全性。

GRIFFIN研究维持治疗阶段，D-R（达雷妥尤单抗/来那度胺）及R（来那度胺）队列的sCR率（62.6％和45.4％）较巩固治疗阶段均明显提升，且D-R对比R队列sCR率的改善更为显著（20.2％对13.4％）[6]。本研究中，达雷妥尤单抗维持治疗使总体sCR率及MRD阴性率分别较治疗前提升了50.0％和33.25％，进一步提高了疗效及缓解深度；同时1q21扩增者sCR率提升了50.0％。MAIA[7]研究显示：D-Rd较Rd方案使1q21扩增者疾病进展风险降低64.0％（*HR*＝0.36，*P*＝0.0008），本研究观察到1q21扩增者的血液学缓解获益。

NDMM伴有重度RI是生存的不利因素，而达雷妥尤单抗方案治疗符合移植条件的NDMM的前瞻性研究均将eGFR<30 ml·min^−1^·（1.73m^2^）^−1^者排除在外[2],[6]，目前该人群达雷妥尤单抗维持治疗的前瞻性数据有限。DARE[8]研究显示：Dd（达雷妥尤单抗/地塞米松）方案治疗复发/难治MM（R/R MM）伴重度RI，在快速获得血液学缓解同时，肾脏缓解率18.4％（肾脏疗效≥PR）。本研究中6例RI-NDMM，1例肾脏疗效MR转为PR，中位eGFR提升24.37％，达雷妥尤单抗维持治疗使RI-NDMM患者的肾功能改善，但改善并不显著，这可能与肾脏损害时间较长，组织结构发生不可逆改变相关。

本研究中位随访12个月，1例死亡（与维持治疗无关），1年OS率93.33％。CASSIOPEIA[2]研究中，VTd（硼替佐米/沙利度胺/地塞米松）序贯auto-HSCT后的观察组中位PFS期为33.6个月，达雷妥尤单抗维持组中位PFS期未达到，疾病进展风险降低68％（HR＝0.32，*P*<0.001）。本研究由于随访时间较短，中位PFS及OS期均未达到，因此需要延长随访时间以观察达雷妥尤单抗维持治疗对于PFS及OS的影响。

达雷妥尤单抗常见的非血液学AE是IRRs，呼吸道症状为其主要表现。SIRIUS[9]研究中IRRs发生率42％，3级发生率5％，96％ IRRs发生于首次输注。本研究中7例（46.67％）发生IRRs，6例发生于首次输注，与SIRIUS研究相近。文献报道，达雷妥尤单抗与上呼吸道细胞表达 CD38 的反应是IRRs原因之一，首次输注前应用孟鲁司特可降低 IRRs率 [10]。

本研究免疫指标监测显示：免疫球蛋白水平于达雷妥尤单抗维持前后均低于正常，且维持治疗后进一步下降。关于免疫球蛋白异常的分析：由于auto-HSCT后体液免疫及细胞免疫重建需要时间，IgG、IgA、IgM分别于auto-HSCT后1个月、12个月、6个月开始恢复[11]。本研究始于auto-HSCT后2～3个月，尚处于体液免疫及细胞免疫缺陷状态；同时达雷妥尤单抗导致浆细胞数减少，使得免疫球蛋白水平进一步下降。

感染是达雷妥尤单抗另一常见AE，以呼吸道感染及肺炎多见。LYRA[12]研究的达雷妥尤单抗维持期，上呼吸道感染发生率：1～2级30.6％，3～4级为0，未发生肺炎；CASSIOPEIA[2]研究维持阶段，3～4级肺炎率3％。本研究3级肺炎5例（33.33％，5/15），其中3例为间质性肺炎，该5例肺炎均为达雷妥尤单抗初治者，肺炎发生时间为达雷妥尤单抗中位输注8次。因此，需要警惕auto-HSCT后的患者本身存在免疫缺陷状态，同时达雷妥尤单抗使体液免疫进一步降低，易导致机会性感染发生；同时亦需警惕达雷妥尤单抗本身及混杂感染所致的间质性肺炎。另一个需要关注的问题：该5例肺炎均发生于达雷妥尤单抗负荷剂量密集给药期，而来自GEN501[13]研究的药代动力学（PK）显示：R/R MM受试者接受每周一次给药，药物清除率随单次剂量增加而降低，当CD38趋于饱和后，靶点清除的影响降至最低；同时药物清除率亦随给药次数增加而下降，可能与肿瘤负荷降低有关；每周16 mg/kg给药，持续8周，可使CD38靶点介导的药物清除率快速饱和[13]。本组研究13例达雷妥尤单抗初治者均为CR或VGPR状态，CD38靶点结合清除对药物清除率影响有限，此时密集增加用药频次可能使药物清除率较R/R MM受试者更快速饱和，继而药物清除延迟，血药浓度相对增高。CASSIOPEIA[2]研究auto-HSCT后维持治疗为每8周一次，即使是达雷妥尤单抗初治者；LYRA[12]研究的维持治疗为每4周一次，以上用药频次均低于本研究，3级肺炎发生率亦减低。同时本组研究中3例间质性肺炎患者，抗细菌、真菌及病毒治疗效果不佳，联合甲泼尼龙治疗有效，此时不能排除免疫因素存在。该3例患者后续每4周一次达雷妥尤单抗维持治疗，未再发生肺炎。当然，以上分析尚不能充分解释本组研究肺炎发生率高的原因，需要了解肺炎的病理改变，结合宿主免疫状态、PK数据等做进一步求证。

综上所述，虽然本研究入组病例数较少，随访时间较短，统计数据较少，但能够观察到达雷妥尤单抗维持治疗使血液学缓解率及缓解深度提升、RI-NDMM患者肾功能改善，且AE可控。本研究旨在为未来扩大样本量深入探讨真实世界符合移植条件的患者达雷妥尤单抗维持治疗的疗效与安全性、维持治疗方案优化等问题提供初始参考数据。

## References

[1] Tacchetti P, Pantani L, Patriarca F (2020). Bortezomib, thalidomide, and dexamethasone followed by double autologous haematopoietic stem-cell transplantation for newly diagnosed multiple myeloma (GIMEMA-MMY-3006): long-term follow-up analysis of a randomised phase 3, open-label study[J]. Lancet Haematol.

[2] Moreau P, Hulin C, Perrot A (2021). Maintenance with daratumumab or observation following treatment with bortezomib, thalidomide, and dexamethasone with or without daratumumab and autologous stem-cell transplant in patients with newly diagnosed multiple myeloma (CASSIOPEIA): an open-label, randomised, phase 3 trial[J]. Lancet Oncol.

[3] Kumar S, Paiva B, Anderson KC (2016). International Myeloma Working Group consensus criteria for response and minimal residual disease assessment in multiple myeloma[J]. Lancet Oncol.

[4] Rajkumar SV (2020). Multiple myeloma: 2020 update on diagnosis, risk-stratification and management[J]. Am J Hematol.

[5] Dimopoulos MA, Sonneveld P, Leung N (2016). International Myeloma Working Group Recommendations for the Diagnosis and Management of Myeloma-Related Renal Impairment[J]. J Clin Oncol.

[6] Voorhees PM, Kaufman JL, Laubach J (2020). Daratumumab, lenalidomide, bortezomib, and dexamethasone for transplant-eligible newly diagnosed multiple myeloma: the GRIFFIN trial[J]. Blood.

[7] Moreau P, Facon T, Usmani S (2022). Daratumumab Plus Lenalid-omide and Dexamethasone (D-Rd) Versus Lenalidomide and Dexamethasone (R-d) in Transplant-Ineligible Patients (Pts) with Newly Diagnosed Multiple Myel-oma (NDMM): Clinical Assessment of Key Subgroups of the Phase 3 Maia St-udy[J]. Blood.

[8] Kastritis E, Terpos E, Symeonidis A (2023). Prospective phase 2 trial of daratumumab with dexamethasone in patients with relapsed/refractory multiple myeloma and severe renal impairment or on dialysis: The DARE study[J]. Am J Hematol.

[9] Lonial S, Weiss BM, Usmani SZ (2016). Daratumumab monotherapy in patients with treatment-refractory multiple myeloma (SIRIUS): an open-label, randomised, phase 2 trial[J]. Lancet.

[10] Moore DC, Arnall JR, Thompson DL (2020). Evaluation of Montelukast for the Prevention of Infusion-related Reactions With Daratumumab[J]. Clin Lymphoma Myeloma Leuk.

[11] 刘 俊茹, 李 娟, 商 京晶 (2013). 多发性骨髓瘤患者自体造血干细胞移植后体液免疫重建及其与感染的关系[J]. 中华血液学杂志.

[12] Yimer H, Melear J, Faber E (2022). Daratumumab, cyclophosphamide, bortezomib, and dexamethasone for multiple myeloma: final results of the LYRA study[J]. Leuk Lymphoma.

[13] Clemens PL, Yan X, Lokhorst HM (2017). Pharmacokinetics of Daratumumab Following Intravenous Infusion in Relapsed or Refractory Multiple Myeloma After Prior Proteasome Inhibitor and Immunomodulatory Drug Treatment[J]. Clin Pharmacokinet.

